# Complete genome sequence of the obligate endosymbiont *Buchnera aphidicola* of the poplar bark aphid *Pterocomma populeum*

**DOI:** 10.1128/mra.00379-25

**Published:** 2025-06-10

**Authors:** Wenjing Sun, Cailing Li, Liyun Jiang, Zhao Pan, Gexia Qiao, Jing Chen

**Affiliations:** 1 State Key Laboratory of Animal Biodiversity Conservation and Integrated Pest Management, Institute of Zoology, Chinese Academy of Sciences 53024 , Beijing, China; 2 Key Laboratory of Zoological Systematics and Application of Hebei Province, School of Life Sciences, Institute of Life Science and Green Development, Hebei University 162640 , Baoding, China; 3 College of Life Sciences, University of Chinese Academy of Sciences 617066 , Beijing, China; The University of Arizona, Tucson, Arizona, USA

**Keywords:** endosymbiotic bacterium, insect, genome sequencing

## Abstract

We report the complete genome sequence of *Buchnera aphidicola* from the aphid *Pterocomma populeum* using Illumina NovaSeq X Plus platform. The assembled genome is 588,150 bp with a guanine-cytosine content of 24%.

## ANNOUNCEMENT

Poplar bark aphid, *Pterocomma populeum* (Kaltenbach, 1843) (Hemiptera: Aphididae: Aphidinae), is a common insect pest on poplar trees. It feeds on branches, 2-year-old twigs, and in bark crevices of *Populus* spp. (1), and can cause shoot dieback under dense colonies (2). This species is distributed in Europe, North Africa, and Asia (1), and has been introduced to North America (3), South America (4), and Australia (5). Aphids feed on phloem sap, which is rich in carbohydrates but low in essential amino acids (6). To compensate for this nutritional imbalance, aphids establish a close symbiotic association with bacteria. *Buchnera aphidicola* is a strictly vertically transmitted, obligate endosymbiont of aphids that provides its host with essential nutrients for development and reproduction (7, 8). Here, we sequenced the genome of *B. aphidicola* from the aphid *P. populeum*.

Samples of *P. populeum* (voucher number 55576) were collected on *Populus* sp. from Aletai, Xinjiang, China (47.79°N, 88.13°E) in July 2022. Total genomic DNA was extracted from whole-body tissues of 10 aphid individuals using the Magnetic Universal Genomic DNA Kit (Tiangen Biotech, Beijing, China). Sequencing libraries of ∼350 bp DNA fragments were prepared using the Watchmaker DNA Library Prep Kit with Fragmentation (Watchmaker Genomics, Boulder, CO, USA), and 150 bp paired-end sequencing was then performed on an Illumina NovaSeq X Plus platform. A total of 15 Gb of raw data were obtained, and 117,746,842 clean reads were generated after removing adapters and low-quality reads in which more than 50% of bases had Q values below 20 or more than 5% of bases were N. We performed assembly using SPAdes v.4.0.0 (9) with the parameters --cov-cutoff auto --isolate -k 21,25,31,33,35,45,55,65,75,77,85,95,105,115,125,127. Next, we aligned the assembled single scaffold to the reference genome of *B. aphidicola* (*Cavariella theobaldi*) (GCF_964059165.1) using MUMmer v.3.2.3 (10), followed by genome polishing with Pilon v.1.23 (11). The genome coverage reaches 292.16×. Assembly completeness was 96.55% as assessed by CheckM v.1.2 (12). Genome annotation was carried out with the National Center for Biotechnology Information Prokaryotic Genome Annotation Pipeline v.6.7 (13).

The complete circular chromosomal sequence of *B. aphidicola* (*P. populeum*) isolate 55576 is 588,150 bp in size, with a guanine-cytosine content of 24%. It contains a total of 578 genes, including 531 protein-coding genes; 10 pseudogenes; 1 each of 5S rRNA, 16S rRNA, and 23S rRNA; 31 tRNAs; and 3 ncRNAs. The newly produced genome of *B. aphidicola* from *P. populeum* provides a valuable data resource for advancing research on *Buchnera* genomics and aphid-*Buchnera* symbiosis.

## Data Availability

The genome sequence of *Buchnera aphidicola* has been deposited in GenBank under accession number CP186013. Accession numbers of the corresponding BioSample and BioProject are SAMN47383379 and PRJNA1236254, respectively. Raw data obtained through Illumina sequencing have been deposited in the National Center for Biotechnology Information Sequence Read Archive database under accession number SRR32698661.
